# Perspectives on the comparative benefits of body-powered and myoelectric upper limb prostheses

**DOI:** 10.1186/s12984-024-01436-4

**Published:** 2024-08-08

**Authors:** Susannah M. Engdahl, Michael A. Gonzalez, Christina Lee, Deanna H. Gates

**Affiliations:** 1 Department of Biomedical Engineering, University of Michigan, Ann Arbor, MI USA; 2 School of Kinesiology, University of Michigan, Ann Arbor, MI USA

**Keywords:** Upper limb, Prosthesis, Control, Rehabilitation, Function, Myoelectric, Body-powered

## Abstract

**Background:**

Patient access to body-powered and myoelectric upper limb prostheses in the United States is often restricted by a healthcare system that prioritizes prosthesis prescription based on cost and perceived value. Although this system operates on an underlying assumption that design differences between these prostheses leads to relative advantages and disadvantages of each device, there is limited empirical evidence to support this view.

**Main text:**

This commentary article will review a series of studies conducted by our research team with the goal of differentiating how prosthesis design might impact user performance on a variety of interrelated domains. Our central hypothesis is that the design and actuation method of body-powered and myoelectric prostheses might affect users’ ability to access sensory feedback and account for device properties when planning movements. Accordingly, other domains that depend on these abilities may also be affected. While our work demonstrated some differences in availability of sensory feedback based on prosthesis design, this did not result in consistent differences in prosthesis embodiment, movement accuracy, movement quality, and overall kinematic patterns.

**Conclusion:**

Collectively, our findings suggest that performance may not necessarily depend on prosthesis design, allowing users to be successful with either device type depending on the circumstances. Prescription practices should rely more on individual needs and preferences than cost or prosthesis design. However, we acknowledge that there remains a dearth of evidence to inform decision-making and that an expanded research focus in this area will be beneficial.

## Background

While the loss of an anatomical hand can have a devastating impact on an individual’s quality of life, upper limb prostheses can help individuals interact more effectively with their environment. In an effort to improve functionality for individuals with upper limb absence, researchers are actively exploring a wide variety of approaches for controlling prosthetic limbs using biosignals acquired noninvasively or invasively from the muscles, peripheral nerves, or brain (see [1] for a detailed review). Many of these approaches show promise for improving functionality but are not yet available in clinical practice. As such, only two options are routinely prescribed today: body-powered (BP) and myoelectric (MYO) prostheses. BP prostheses are biomechanically powered (i.e., shoulder and/or trunk movement is translated to terminal device actuation via a harness and Bowden cable), while MYO prostheses are externally powered using electrical activity recorded from residual limb musculature. Although a variety of different terminal devices, socket designs, and other components may be used to construct either type of device, BP and MYO prostheses are viewed as conceptually distinct treatment categories due to their different underlying control modalities.

This differentiation between BP and MYO prostheses based on their control modalities might suggest that each design offers relative advantages compared to the other and thus should be considered equally valid options for patient treatment. However, this view is not widely accepted within the United States healthcare system, where prostheses are viewed on a hierarchy of complexity and value [2]. Under this model, insurance policies may prioritize more “basic” technologies (i.e., BP prostheses) in favor of more “advanced” technologies (i.e., MYO prostheses). This tiered categorization implies that BP devices should be considered the standard of care, leading to the implementation of policies in which MYO prostheses are completely excluded from coverage or covered only if BP devices are shown to be insufficient for supporting a patient’s functional goals [3–6]. These policies may contribute to health disparities based on an individual’s ability to pay for their care, making certain prosthetic technologies unavailable to those who are financially disadvantaged [7]. Notably, one survey found that 48% of individuals with upper limb absence who do not use a prosthesis cited cost as a major reason for this choice [8].

Setting aside any practical constraints imposed by payer restrictions, prosthesis prescription practices would ideally be guided by a determination of what is best for each individual patient based on a wide variety of considerations. However, there is limited empirical evidence available to direct these decisions. This point is demonstrated by two systematic literature reviews which identified 27 experimental studies comparing BP and MYO prostheses published through 2016, of which only 11 used functional or laboratory-based assessments. These reviews failed to substantiate whether BP or MYO prostheses provide a significant general advantage over the other based on this small body of evidence [9, 10]. Similarly, the 2022 VA/DoD Clinical Practice Guideline for the Management of Upper Extremity Amputation Rehabilitation states that there is insufficient evidence to recommend “any specific control strategy, socket design, suspension method, or component” [11] due to low confidence in the quality of existing evidence. Moreover, a clinical practice guideline for prosthetic management of transradial limb absence was developed in 2021 through a consensus process among subject matter experts, rather than empirical support [12]. This lack of quantitative data may explain why prescription practices are instead motivated by factors such as prosthetist experience, patient input, manufacturer claims, and payer restrictions [9]. It may also create barriers to advancing research on prosthesis design and control, as it can be unclear where to prioritize efforts for improvement.

This commentary article will summarize a collection of studies that we recently completed to compare functional task performance between BP and MYO prosthesis users. While many evidence gaps still remain to be filled, we offer some preliminary observations based on our work in this area. We hope that these perspectives will motivate additional studies to gather evidence that informs appropriate clinical prescription guidelines and insurance reimbursement policies, which is crucial for advancing the quality of care available to individuals with upper limb loss.

### Note on methodology

The studies referenced in this commentary involved nine adult BP or MYO prosthesis users with transradial limb loss or congenital transradial limb absence (Table 1). Three of these participants (P01, P07, and P08) owned both BP and MYO prostheses. They were tested using both prostheses when possible, although P07 and P08 only used their BP prostheses during some studies due to time constraints or malfunctions in their MYO prosthesis (i.e., P07 did not complete studies [13–15] with his MYO prosthesis and P08 did not complete study [16] with his MYO prosthesis). The terminal devices included voluntary-open split hooks, voluntary-close split hooks, single degree-of-freedom hands, and multi-articulated hands. Note that participants who owned multi-articulated hands used standard direct control strategies, rather than pattern recognition. Unless indicated otherwise, all results presented below have been previously published.

**Table 1:** Characteristics of study participants

ID	Cause of limb absence	Prosthesis type	Terminal device	Time since amputation	Duration of prosthesis ownership
P01	Acquired	BP	Voluntary-open split hook	14 months	10 months
		MYO	Multi-articulated hand (iLimb)		5 months
P02	Acquired	BP	Voluntary-open split hook	9 months	7 months
P03	Congenital	MYO	Single degree-of-freedom hand	n/a	33 years
P04	Congenital	MYO	Multi-articulated hand (bebionic)	n/a	6 months
P05	Congenital	MYO	Multi-articulated hand (bebionic)	n/a	10 months
P06	Acquired	BP	Voluntary-open split hook	2.5 years	2 years
P07	Acquired	BP	Voluntary-open split hook	10 years	7 years
		MYO	Multi-articulated hand (iLimb)		2 years
P08	Acquired	BP	Voluntary-close split hook	24.75 years	23 years
		MYO	Single degree-of-freedom hand		23 years
P09	Acquired	BP	Voluntary-open split hook	11 months	4 months

### Impact of prosthesis design on multisensory integration

Given the dissimilarities in their respective methods of actuation, BP and MYO prostheses offer different interfaces through which users can interact with the surrounding environment. In particular, these actuation methods might affect a user’s ability to detect and interpret sensory feedback, which is significant since prostheses inherently lack much of the sensory feedback available to an individual using their anatomical hand. For example, when using a BP prosthesis, control of the terminal device is directly coupled to movement of the scapula via a Bowden cable. This direct transfer of position and, in some cases, force feedback results in a sense of position and effort at the terminal device, a phenomenon called extended physiological proprioception. Other available sources of feedback can include forces transmitted through the prosthesis socket, as well as sound or vibration (which may either be artificially provided or natively available from other sources, such as the motors in a MYO prosthesis) [17, 18]. However, these feedback sources are often inaccessible or insufficient for MYO users, so they instead rely extensively on vision during active grasping and manipulation [19, 20]. Given that multiple domains of motor behavior are dependent on the availability and interpretation of sensory feedback, we hypothesized that differences in behavior would be detectable based on whether a BP or MYO prosthesis was used. Further consideration of these domains will be presented subsequently, including identification of object properties, embodiment of the prosthetic limb, movement quality, and overall kinematic patterns.

While differences in sensory feedback between prosthesis types have been described in the literature through anecdotal evidence [9], no studies have empirically compared feedback available to people with upper limb absence using their own prostheses. To address this gap, we asked participants to complete grasping tasks with their prescribed prosthesis and anatomical limb under different feedback conditions using a custom haptic device [13]. In a grasp aperture matching task, participants attempted to close their hand or terminal device to a specific width based on a visual target. Their hands were hidden from view, and depending on the condition, they were provided with additional visual, vibrotactile, or force feedback. They also completed the task without any additional feedback. In a stiffness identification task, participants probed a virtual object and were asked to determine if it had a low, medium, or high stiffness. Participants had to determine object stiffness using either only visual or only force feedback.

**Fig. 1:**
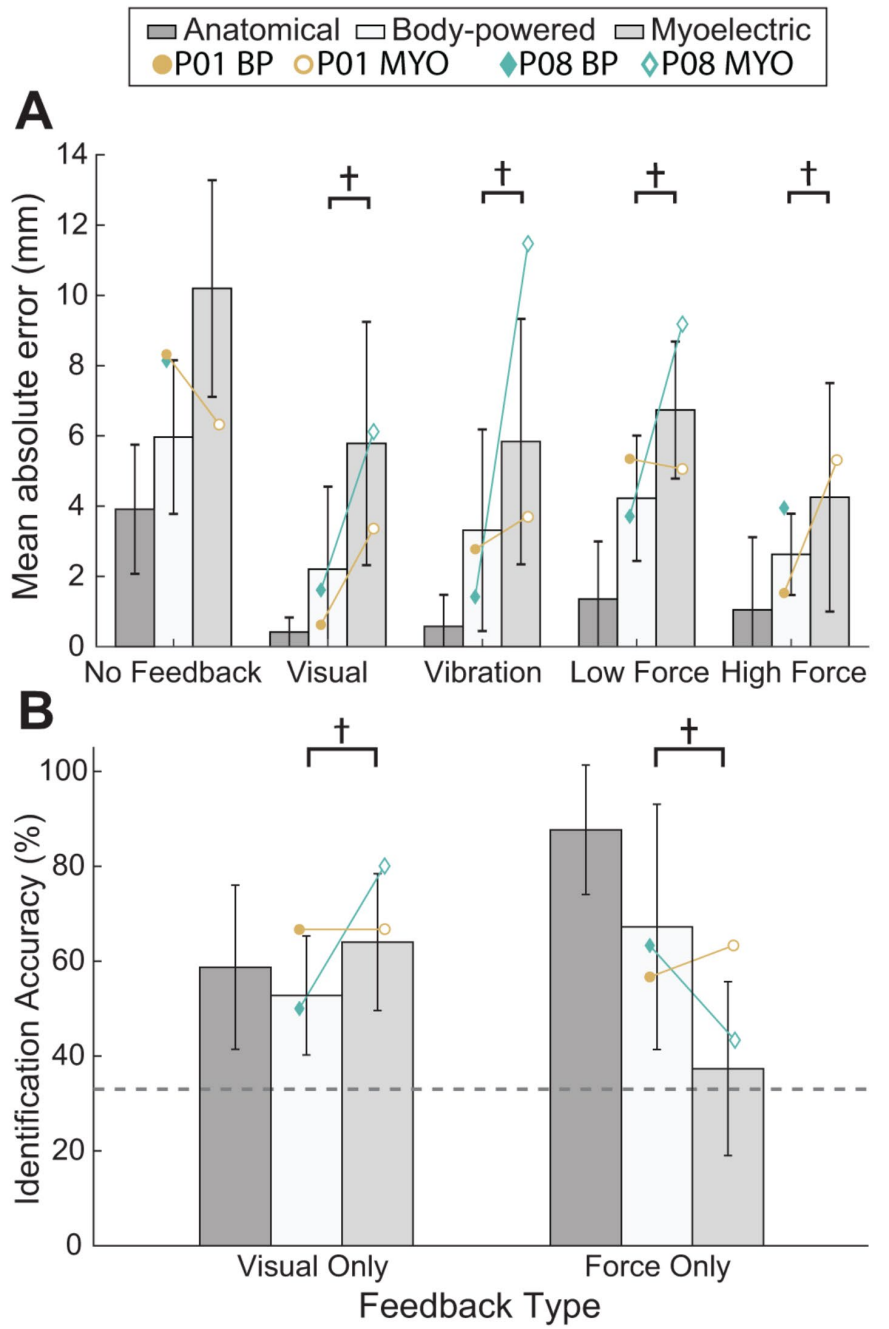
(**A**) Mean absolute error between paddle position and target position for the aperture matching task. (**B**) Identification accuracy of blocks during the stiffness identification task. The dotted line represents the level of chance for the task. Within-subject means for P01 and P08 are displayed in both plots as open circles or diamonds, respectively. Bars represent the average for each group. ‘†’ indicates medium or greater Hedges’ *g* effect size (g ≥ 0.5) for the pairwise comparison between BP and MYO prostheses

BP users were able to utilize force feedback to a greater extent than MYO users in both the aperture matching (Fig. 1A) and stiffness identification (Fig. 1B) tasks [13]. While being provided with force feedback did decrease aperture errors for MYO users, they performed close to chance when identifying object stiffness. The difference in tasks may be due to transfer of forces through the prosthesis socket, which is more noticeable for acute changes like contacting an object, compared to continuous changes as when probing an object to gauge stiffness. In support of the idea that MYO users rely primarily on vision, MYO users were marginally more accurate at identifying object stiffness using only visual feedback compared to BP users. Notably, there was considerable variance across participants, suggesting that there are potentially more factors than device design that are important for interpreting feedback. For example, P08 had the longest duration of prosthesis ownership among participants and was more accurate at identifying object stiffness with his MYO prosthesis using visual feedback compared to most other participants using visual feedback, regardless of limb type (Fig. 1B).

The extent to which a prosthesis user can detect and interpret sensory feedback may have additional consequences beyond their ability to accurately identify object properties. For instance, embodiment is generally considered to be a body representation or phenomenological perception that incorporates a prosthetic limb (e.g. the prosthesis becomes part of the body representation or is perceived to be part of the body) [21]. An individual’s development of prosthesis embodiment is thought to depend on a combination of multisensory integration, volitional intent, and dynamic interaction with the environment [22]. In particular, there needs to be congruence between tactile, visual, and proprioceptive signals [23] that are concordant with the user’s volitional control over the prosthesis. Given that prosthesis embodiment is related to the integration of multisensory inputs, differences in the availability of sensory feedback between BP and MYO prostheses could mean that the extent of embodiment differs between prosthesis designs as well. Specifically, if BP prostheses offer inherent proprioceptive feedback that MYO prostheses do not, this could suggest that BP prostheses may be embodied more strongly.

**Fig. 2:**
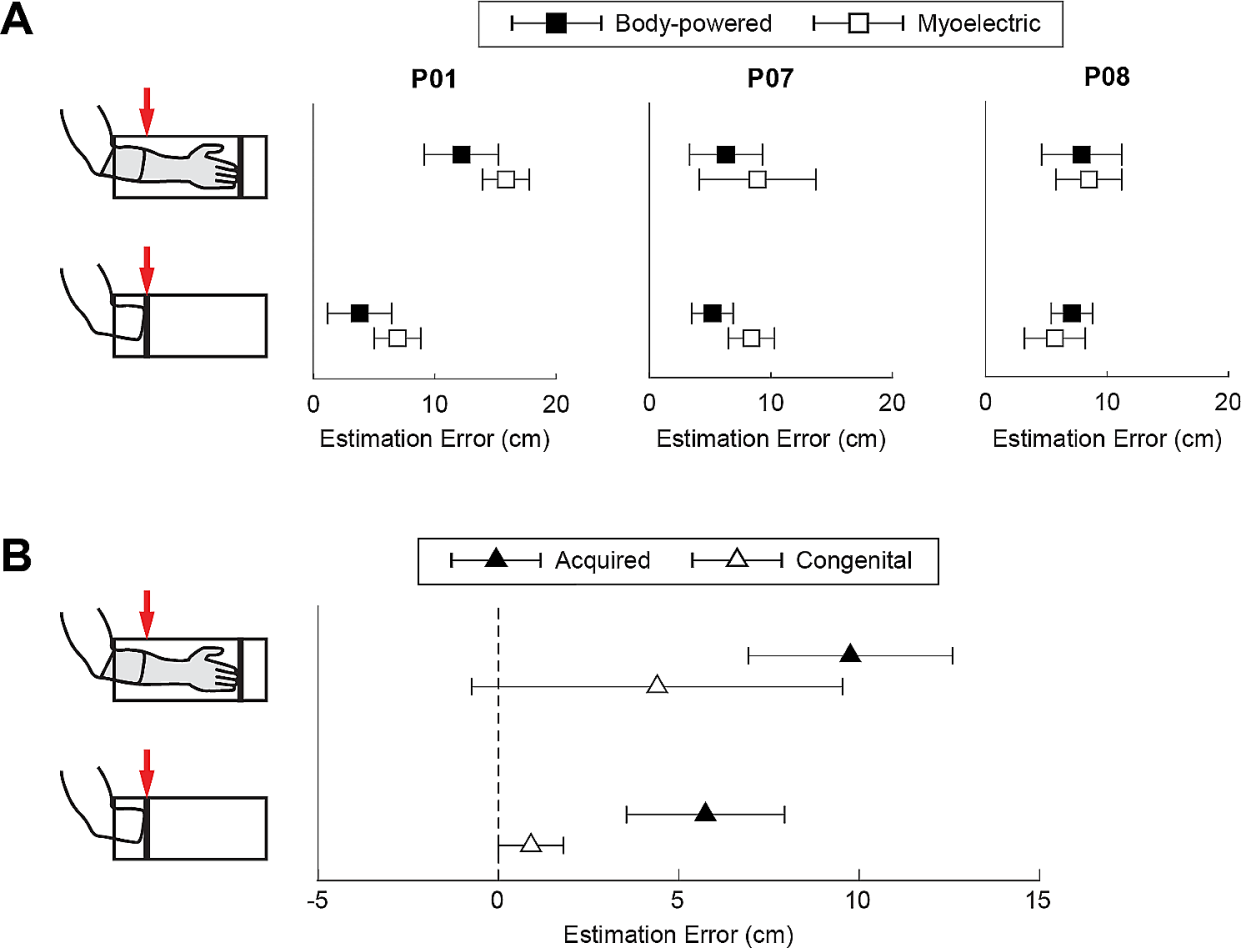
Participants performed the residual limb length estimation task by placing either their prosthetic limb or residual limb inside an opaque tube and estimating where they perceived the end of their residual limb (red arrows). Positive estimation error indicates overestimation of residual limb length. (**A**) Average limb length estimation error for participants who had both BP and MYO prostheses. (**B**) Average limb length estimation error for participants who had acquired limb loss and congenital limb absence

To test this hypothesis, we asked prosthesis users to complete surveys about perceived prosthesis ownership and agency, as well as a residual limb length estimation task focused on perceptual adaptations to limb absence and prosthesis use [24]. In this paradigm, overestimation of residual limb length while wearing a prosthesis indicates that the user’s perception of their residual limb has expanded into the space occupied by the prosthesis. This overestimation may be viewed as a metric of embodiment [25] (although it is by no means the only option for measuring embodiment [21]). Residual limb length estimation was also performed without the prosthesis to indicate whether this overestimation is retained when the prosthesis is removed. None of these outcomes significantly differed between BP and MYO prosthesis users, as there was significant variability in responses. Even within the three participants who used both BP and MYO prostheses, results were inconsistent (Fig. 2A). This suggests that other participant factors may contribute to embodiment. Here, we found several significant trends that were driven by participant characteristics other than prosthesis type. One influential characteristic was cause of limb absence (acquired vs. congenital). For example, participants with acquired limb loss tended to overestimate their residual limb length both with and without the prosthesis, but participants with congenital limb absence estimated more accurately (Fig. 2B). Additionally, greater residual limb length estimation error when not wearing a prosthesis was correlated with increased hours of daily prosthesis wear. Collectively, our results could support the hypothesis that prosthesis embodiment is also dependent on an individual’s experiences with limb loss and prosthesis use, not just the features of their prosthesis. If this conjecture is validated in future work, it might de-emphasize the need to consider prosthesis design or control strategy as the primary means to promote embodiment.

### Impact of prosthesis design on movement planning and performance

Building on the idea that the user-prosthesis interface varies between BP and MYO prostheses based on their methods of actuation, it may also be possible that prosthesis design would impact how users plan and execute functional movements. Reaching movements rely on the ability to plan movements using an internal model that is dependent on the awareness of upper limb properties (i.e., feedforward control) and the ability to make movement corrections by incorporating various forms of environmental feedback, such as vision and tactile sensation (i.e., feedback control). In a healthy limb, these control systems are used to produce reaching movements characterized by features such straight position trajectories with symmetric velocity profiles [26], blended sub-movements [27], and temporal coupling between reaching and grasping [28]. If these control systems are impaired in prosthesis users, it may result in abnormal movement patterns.

For example, individuals using their anatomical limbs are able to efficiently adapt to environmental changes and maintain accuracy in their reaching movements. However, using a prosthesis alters the inertial properties of the limb, requiring the formation of an updated internal model in order to perform accurate movements. Previous studies have indeed found that prosthesis users can complete accurate goal-directed reaching movements while adapting to force field perturbations [29] and changes in visual feedback [30] to a similar extent as those without amputation, although with lower peak speeds required to achieve similar task accuracy [30]. The potential effects of prosthesis type on these outcomes have not been explored in detail, however.

**Fig. 3:**
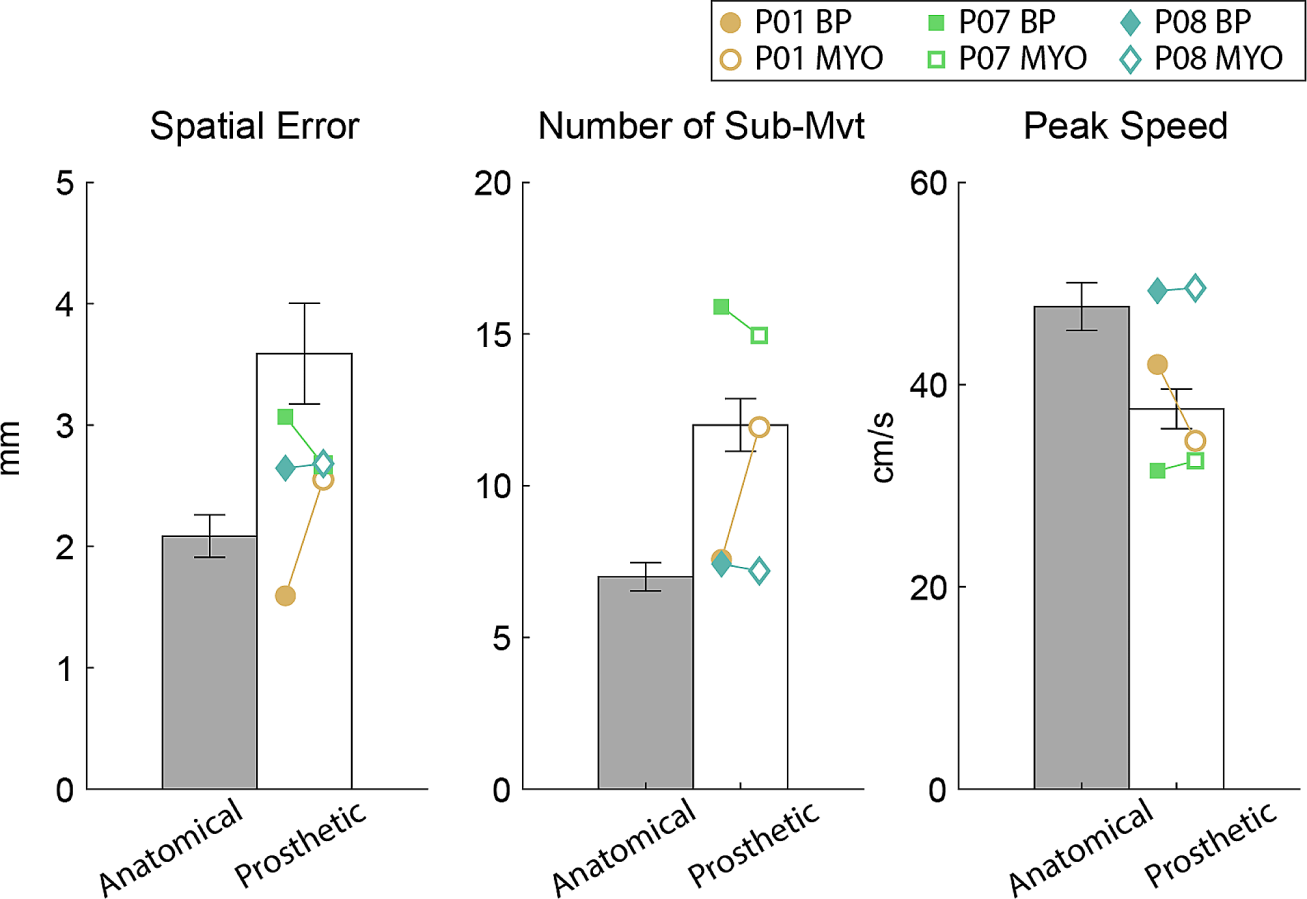
Average (bar) and standard error (error bar) of spatial error (mm), number of sub-movements, and peak speed (cm/s) of movements completed by both anatomical limbs of nine control participants (grey) and prosthetic limbs of nine individuals with limb absence (white). Decreased number of sub-movements and increased peak speed represent increased movement quality. Within-subject means for P01, P07, and P08 are shown as individual markers

Towards this end, we compared movement accuracy during goal-directed planar reaching between prosthesis users and individuals without limb absence [16]. The reaching tasks were designed with either a spatial or temporal goal. During the spatial task, the participants completed mediolateral reaching movements with the goal of aligning the handle position to the position of a target on a screen as accurately as possible. During the temporal task, participants moved the handle back and forth mediolaterally, in time with a metronome. Movements made with anatomical limbs and prosthetic limbs were completed with similar accuracy during ballistic movements that primarily relied on feedforward control (temporal task), suggesting that prosthesis users were able to accomplish the task with a sufficient internal model. However, movements with prosthetic limbs were completed with reduced end-point accuracy during a spatial reaching task that required substantial feedback control. To supplement our published results, here we conducted an exploratory analysis of the three participants who had both BP and MYO prostheses. While these results are descriptive and not supported with statistical analysis, it is anecdotally apparent that differences between the devices were not consistent for these individuals. Specifically, P01 and P08 completed the task with lower endpoint error with their BP prostheses, while P07’s movement was more accurate when using a MYO prosthesis (Fig. 3).

Another way to characterize healthy motor behavior is through measurement of movement smoothness [31]. Prior studies suggest that movement quality (i.e., smoothness) is generally lower for prosthetic limbs than anatomical limbs [32–35], likely due to challenges with the feedback control systems. However, movement quality could also differ between BP and MYO prostheses based on how users interface with each type of device. For example, greater dependence on visual feedback in MYO prosthesis users might result in reduced movement quality compared to BP prosthesis users. Moreover, movement quality might be influenced by the level of effort required to open and close the terminal device, such that the mechanical actuation of BP prostheses could lead to reduced movement quality on tasks that require significant terminal device movement. We explored this issue during both constrained planar reaching without terminal device actuation and during unconstrained reaching and object manipulation.

During constrained planar reaching (i.e., the spatial task described previously), we quantified movement quality measures (number of sub-movements and peak speed) as a supplemental descriptive analysis in three participants from [16] who owned both types of prostheses. We found no consistent differences in outcomes based on prosthesis type in these individuals (Fig. 3). P01 completed the task with more sub-movements and lower peak speed with his MYO prosthesis, while P07 and P08 had better movement quality with their MYO prostheses. Interestingly, P08 had the most similar duration of prosthesis ownership between two devices (23 years for both) and exhibited fewer differences in movement quality compared to the two other participants who had varying differences in the duration of ownership (5 months and 5 years, respectively). Therefore, it is possible that the duration of ownership may play a role in the quality of goal-directed movements.

**Fig. 4:**
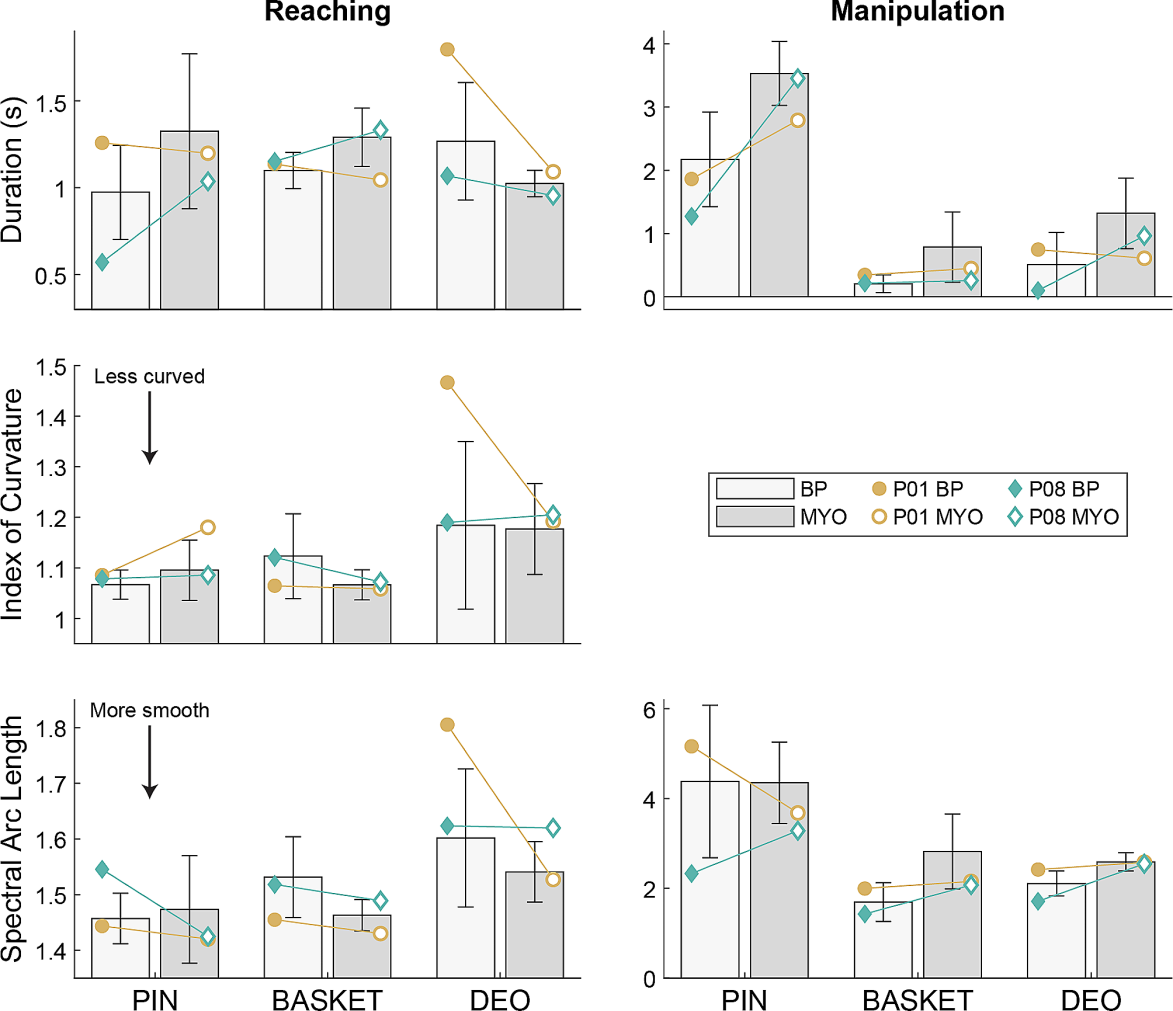
Average (bar) and standard deviation (error bar) of movement quality metrics achieved during representative unilateral (PIN; place pin in corkboard), symmetrical bilateral (BASKET; lift basket from floor to table), and asymmetrical bilateral (DEO; apply deodorant) ADLs by BP and MYO prosthesis users. Within-subject means for P01 and P08 are shown as individual markers

During unconstrained activities of daily living (ADLs) that required reaching and object manipulation (i.e., grasping and/or releasing), we quantified duration, straightness, and smoothness as metrics of movement quality [14]. Movement quality was similar between the two prosthesis types during reaching, with a few exceptions. Movements with BP prostheses were slower and less smooth when reaching to a deodorant stick and movements with MYO prostheses were slower when reaching to place a pushpin on a corkboard. During object manipulation, movements with MYO prostheses were typically slower and less smooth than those with BP prostheses, but these differences were not present for all tasks. Comparison of the two individuals who used both BP and MYO prostheses on a subset of ADLs shows that they usually, but not always, followed the group-level trends (Fig. 4). Additionally, the magnitude and/or direction of the change in outcome measures between devices sometimes differed between participants. For example, P08 moved more slowly during the reaching phase of a pushpin task with his MYO prosthesis compared to his BP prostheses (following the group trend), but P01 moved more quickly with his BP prosthesis compared to his MYO prosthesis. This variance in the results based on task, movement phase, and individual could suggest that neither prosthesis type offers an absolute advantage in terms of movement quality.

Another way in which the actuation method of a prosthesis might affect how users execute functional movements relates to their overall kinematic patterns. Many prostheses, both BP and MYO, are limited to a single degree of freedom (e.g., open/close) in their terminal device. Without the ability to control more distal degrees of freedom, prosthesis users often perform compensatory strategies like altering the trunk, shoulder, or elbow range of motion used by the side of their body with limb absence [36–41]. Differences in how the terminal device is actuated might translate to unique patterns of compensatory movement based on which type of prosthesis is used. For example, the shoulder and trunk movement involved with operating a BP prosthesis may induce different compensations compared to operating a MYO prosthesis. The suspension mechanism typically involved with a MYO prosthesis may also impact the elbow range of motion in users.

We quantified upper limb and trunk range of motion in BP and MYO prosthesis users during ADLs that required reaching in different planes and manipulating objects with various sizes and shapes [15]. Participants used greater trunk lateral lean during deodorant application when using a BP prosthesis compared to a MYO prosthesis. Additionally, BP users had greater trunk axial rotation and lower shoulder elevation relative to MYO users when placing a box on a high shelf. Otherwise, range of motion did not significantly differ based on prosthesis type. The observed differences may be attributed to reduced shoulder mobility from the harnesses of BP prostheses, which decreases the wearer’s reachable workspace [42, 43] and necessitates increased trunk motion to orient the arm during task performance.

**Fig. 5:**
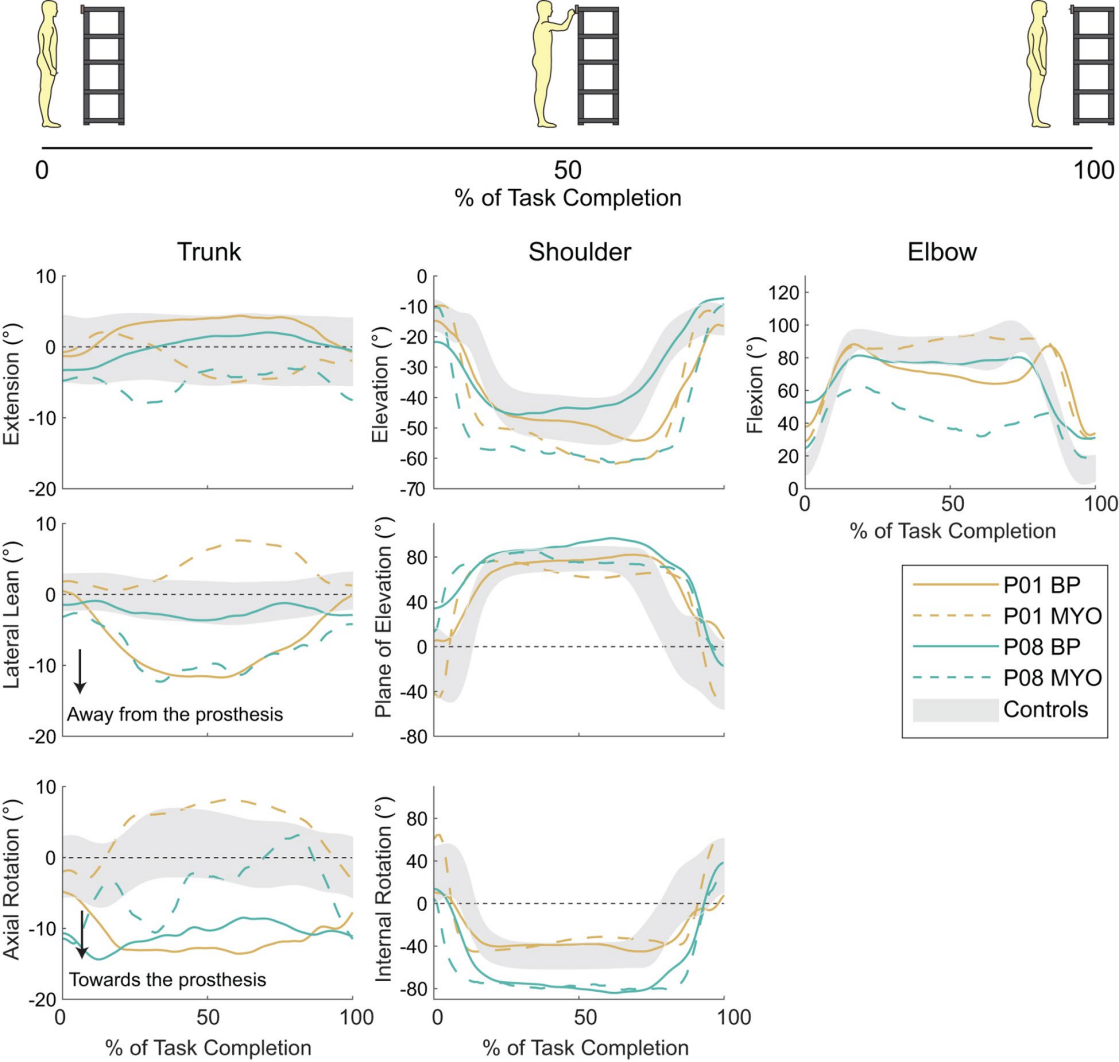
For an ADL involving placing a pin on a corkboard, we qualitatively compared trunk and upper limb angle trajectories for P01 and P08

Other insights were gleaned by directly comparing two individuals who used both BP and MYO prostheses. These individuals varied greatly in the kinematic patterns used during certain ADLs based on which type of prosthesis was used (≥ 10º difference on some joint angles). However, the specific ADLs and joint angles for which their patterns differed were inconsistent between the individuals. For example, when pushing a pin into a corkboard (Fig. 5), P01 used similar peak elbow flexion angles with both prostheses but P08 used much higher peak elbow flexion angles with their BP prosthesis compared to their MYO prosthesis. Similar inconsistencies were found for other ADLs (see Appendix 5 in [15]). Although compensatory movements were not systematically correlated with duration of prosthesis ownership, socket comfort, or terminal device type, it is possible that compensatory movements would be affected by other personal characteristics beyond these.

**Fig. 6:**
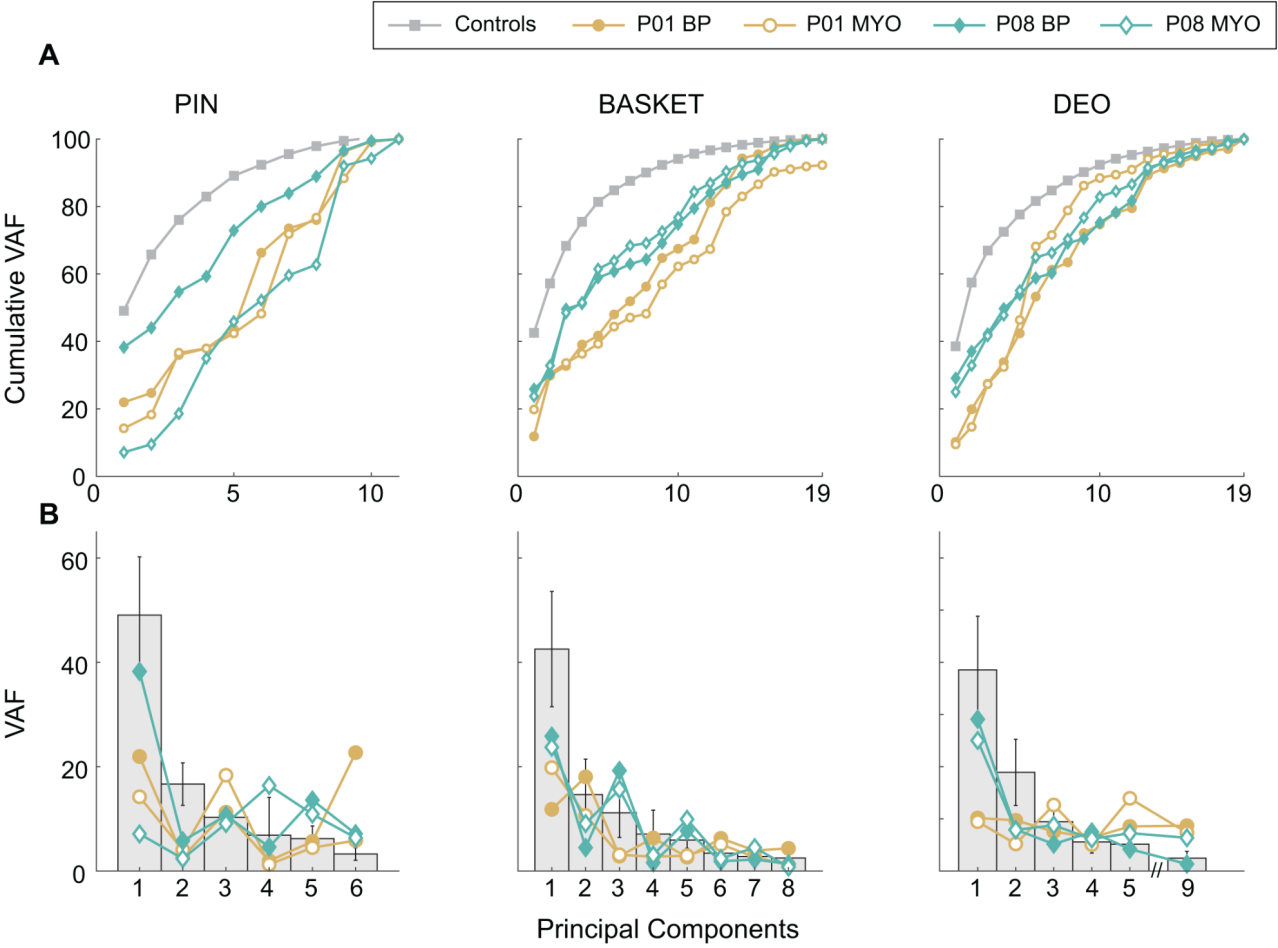
(**A**) Cumulative VAF and (**B**) VAF of individual principal components during movements involving a prosthesis during representative unilateral (PIN; place pin in corkboard), symmetrical bilateral (BASKET; lift basket from floor to table), and asymmetrical bilateral (DEO; apply deodorant) ADLs by P01 (yellow circles) and P08 (cyan diamonds). Movements involving a BP and MYO prosthesis were represented by solid markers and open markers. Average cumulative VAF and VAF of individual principal components during anatomical movements completed by control participants are plotted with grey squares and grey bars for reference

While the joint range of motion quantifies the extent of compensatory movement needed to accomplish a task, it does not measure how different joints work together. To address this, we evaluated inter-joint coordination strategies in prosthesis users during ADLs [44] using principal component analysis with the trunk and upper extremity joint trajectories as input features. Principal components are considered coordinative units that reveal how different joint trajectories are organized. These principal components were obtained by calculating a common set of weighting coefficients for each ADL from concatenated control participants’ data (i.e., movements made by individuals without limb loss were used for a baseline reference). As a measure of inter-joint coordination for participants performing each ADL, we then quantified the cumulative percent of variance accounted for (VAF) by the first five principal components. In other words, the VAF indicates the extent to which the movements were organized similarly to movements made by individuals without limb loss. Our results showed that prosthesis users coordinated their movements differently during each ADL (i.e., reduced VAF) when compared to movements completed by control participants. As a separate part of this commentary, we also preliminarily explored device effects for P01 and P08, as they completed ADLs with both BP and MYO prostheses (Fig. 6). Similar to range of motion, the differences in cumulative VAF between prosthesis types within each participant were not consistent. Out of five ADLs, the only consistent differences between devices for P01 and P08 were a greater cumulative VAF with their BP prosthesis when placing a pushpin in corkboard and with their MYO prosthesis when applying deodorant. Therefore, it is unlikely that a specific prosthesis type provides comparative benefits in prosthesis users’ inter-joint coordination.

## Discussion

The series of studies outlined in this paper was intended to advance the knowledge base surrounding functional differences in BP and MYO prosthesis use. As identified in prior reviews, there is a shortage of quantifiable evidence from which to draw conclusions on this issue [9, 10]. Although these studies have only marginally expanded the evidence, we believe that they may still prove useful in developing future guidelines for prosthesis prescription and insurance reimbursement policies. Existing evidence highlights the limitations in viewing upper limb prostheses on a hierarchy of value and suggests the need for using different approaches towards comparative studies of prosthesis use.

While it seems reasonable to hypothesize that functional differences in BP and MYO use might arise from differences in actuation mechanism, this hypothesis is not currently supported by the literature. For example, a literature review showed low levels of evidence gleaned from expert opinions to support that BP prostheses provide more sensory feedback than MYO prostheses [9]. While Gonzalez et al. [13] demonstrated that BP users could use sensory cues to more accurately match a target terminal device aperture compared to MYO users, users of either device could judge object stiffness similarly as long as visual feedback was present. Furthermore, functional differences based on prostheses type were either absent or inconsistent and seemed to depend more on the particular task being tested or patient-specific characteristics (e.g., cause of limb absence). This aligns with prior reports that there is insufficient evidence to indicate that either BP or MYO prostheses offer absolute advantages over the other system [9, 10].

There may be many potential causes for the lack of evidence regarding difference between prosthesis types. Limitations in study design may be the simplest explanation, as the considerable heterogeneity between participants combined with small sample sizes can make it challenging to find statistically significant trends. This is a well-known challenge in upper limb prosthesis research. A contrary view is that trying to control for inter-subject variability in experimental design may obscure important insights, as this variability may be the key to improved understanding of patient functional outcomes. Thus, we may be unable to identify a single factor, or even a small number of factors, that predict patient success with a prosthesis. In this case, the field would be better served by focusing future efforts on exploring which prosthesis is appropriate for each individual patient given their unique combination of needs, preferences, medical history, and psychosocial characteristics. Alternative study designs, like within-subjects comparisons or small-n studies, may be better equipped to explore this idea. Beyond these patient-specific factors, prosthesis training is also associated with increased prosthesis use [45] and satisfaction [46]. Because few clinicians specialize in treating upper limb loss [47] and evidence-based guidelines for training prosthesis use are lacking [48], training can vary considerably both in duration and technique. We did not collect information about prosthesis training in our studies and cannot comment on how it might have affected each participant’s performance, but we encourage deeper consideration of how training influences patient outcomes in future work.

In addition, it is important to remember that indicators of “patient success” are not universal. For example, cosmesis may be the highest priority for some users—in which case, biomechanical outcome measures (e.g., movement quality) would be less critical for demonstrating successful adoption of their prosthesis. Regardless of which outcomes are identified as the most important for a specific user, those metrics might not actually be relevant to healthcare policies in the United States. These policies are informed, at least in part, by the established set of outcomes available for demonstrating value in providing patients with a prosthesis. Since most existing clinical evaluations rely on expert observation or time-based tests, those particular outcomes play a substantial role in determining what a policy will cover. Regrettably, these evaluations fail to capture subtleties in user performance related to movement quality or overall kinematic patterns, as biomechanical outcomes cannot be quantified through visual inspection alone and may only correlate with a limited range of clinical test scores [41, 49, 50]. Current clinical evaluations also do not obviously address more foundational user experiences like embodiment or integration of sensory feedback. If these outcomes are more conclusively shown in future work to be relevant for characterizing individual user performance, it will be critical to include them in clinical assessments as a way to reshape policy priorities.

It is interesting to note that although we saw differences in usability of sensory feedback based on prosthesis design, it did not translate to consistent functional differences in other domains like embodiment or movement quality. One explanation is that studies of prosthesis users’ ability to access and interpret sensory feedback have been conducted in highly controlled settings, which removed many complicating factors that are encountered in real-world prosthesis use. While these studies may reflect some basic trends in how an individual’s feedback or feedforward control systems interact with a prosthesis design, these fundamental motor behaviors may be outweighed in practice by factors like task requirements. This may explain why the studies of higher-level function during ADLs failed to show consistent differences in movement quality or movement patterns. Relatedly, it may be useful to more directly quantify whether these outcomes are interrelated. For example, measures of embodiment could be correlated with “downstream” biomechanical outcomes (e.g., correlation between embodiment and reaching duration in [51]). This might help highlight trends based on individual patient characteristics that were hidden in the group level comparisons performed in our prior studies. Additionally, studies of real-world prosthesis use may offer insight beyond traditional lab-based studies (e.g., [52, 53]).

More broadly, we would like to emphasize the general need for continued research on BP and MYO prostheses with standard direct control strategies. Considerable research effort is being focused on developing new technologies and algorithms to more effectively derive prosthesis control commands from a variety of biosignal sources, expanding the user’s ability to monitor their environment through sensory restoration, and mechatronic development of highly dexterous prosthetic componentry, among other areas [1]. While such development is necessary to advance patient care in the long run, it requires significant financial expense in the United States and many years of development to bring new products to market. Furthermore, there is no guarantee that new products will be granted an L code in the Healthcare Common Procedure Coding System (HCPCS), which can limit or prohibit product reimbursement from health insurance providers and impede a patient’s access to that product. As such, there is a need to make sure patients have access to quality care through existing technologies [54] and the improvement of these technologies should not be neglected. BP prostheses in particular have received remarkably little attention in the literature [55] despite their continued applicability to clinical care. Prosthesis users have identified features including reduced weight, improved durability, more effective temperature/perspiration management, more comfortable harness/strap systems, and reduced noise as some of their priorities for device improvement. While these topics are somewhat outside of the currently prevalent research areas identified above, they nonetheless stand to have a substantial impact on patient care.

Moreover, BP and direct control MYO prostheses are unlikely to become irrelevant even when new technologies come to market, as they are well-established options that can lead to positive functional outcomes for many patients. In fact, patients may prefer to continue using these options due to familiarity, satisfaction with current functionality, or inability to access newer technologies that are more expensive. Our prior survey studies found that even though individuals with upper limb absence support the development of more advanced technology, many remain interested in using traditional prostheses [56, 57]. Comments from several of these individuals revealed frustration at the lack of research focused on improving these devices [56]:

*“Why can’t anyone just make body powered prostheses and traditional suspensions a little bit better instead of trying to do all of this stuff that’s too expensive and hard to create? If we had spent just a fraction of the hundreds of millions of dollars spent on brain controlled monkey research on improving the basics*,* maybe we’d actually have had a decent new prosthetic arm hit the market in the last 10 years.”*

*“A lot of emphasis continues to be placed on R&D for external powered solutions to upper extremity prostheses. Body powered prostheses and activity specific prostheses R&D get almost no support. Both technologies should be developed and researched.not just “bionic” technology…Pursue the research but balance the research with also a pursuit of improving other more basic and more functional*,* less expensive reliable technologies.”*

This is not to say that emerging technologies should be discounted as part of ongoing research efforts to understand the relative advantages of different prosthesis designs. For example, the use of MYO pattern recognition as an alternative to direct control has become increasingly common, with several commercialized systems now available for clinical use. While these systems are still used less frequently than their more well-established counterparts (i.e., BP and direct control MYO), they are prevalent enough to merit consideration. We did not focus on pattern recognition in our studies since our MYO users only had direct control systems, so our conclusions should be interpreted with that limitation in mind. It is possible that pattern recognition systems would yield more consistent outcomes across individuals or more substantial differences when compared to BP systems than we found in our work focused on direct control systems. Thus, research including MYO pattern recognition is needed for the development of more informed, patient-focused healthcare policies that can provide patients with the most appropriate prosthesis system to meet their individual needs.

## Conclusion

There is currently insufficient evidence to demonstrate whether BP and MYO upper limb prostheses consistently offer relative functional advantages over each other solely due to device design. We speculate that performance is instead more dependent on the specific tasks involved and individual user characteristics, such that prosthesis users can be successful with either type of device depending on the circumstances. Thus, prosthesis prescription might be more appropriately driven by a consideration of individual needs and preferences rather than a perception of value based on device design. However, this hypothesis is drawn from a small body of evidence and cannot be substantiated without additional exploration. More studies of individual user performance with upper limb prostheses across a variety of domains are needed to advance the development of evidence-based prescription practices.

## Data Availability

The datasets referenced within the article are available from the corresponding author on reasonable request.

## Abbreviations

ADL: Activity of daily living
BP: Body-powered
HCPCS: Healthcare Common Procedure Coding System
MYO: Myoelectric
VAF: Variance accounted for
